# Mesolimbic Neurobehavioral Mechanisms of Reward Motivation in Anorexia Nervosa: A Multimodal Imaging Study

**DOI:** 10.3389/fpsyt.2022.806327

**Published:** 2022-03-07

**Authors:** Reza Tadayonnejad, DS-Adnan Majid, Evangelia Tsolaki, Riddhi Rane, Huan Wang, Teena D. Moody, Wolfgang M. Pauli, Nader Pouratian, Ausaf A. Bari, Stuart B. Murray, John P. O'Doherty, Jamie D. Feusner

**Affiliations:** ^1^Division of Neuromodulation, Semel Institute for Neuroscience and Human Behavior, University of California, Los Angeles, Los Angeles, CA, United States; ^2^Division of Humanities and Social Sciences, California Institute of Technology, Pasadena, CA, United States; ^3^Cognitive Neuroscience, Semel Institute for Neuroscience and Human Behavior, University of California, Los Angeles, Los Angeles, CA, United States; ^4^Department of Neursurgery, University of California, Los Angeles, Los Angeles, CA, United States; ^5^Artificial Intelligence Platform, Microsoft, Redmon, WA, United States; ^6^University of Texas Southwestern Medical Center, Dallas, TX, United States; ^7^Department of Psychiatry and the Behavioral Sciences, University of Southern California, Los Angeles, CA, United States; ^8^Computation & Neural Systems Program, California Institute of Technology, Pasadena, CA, United States; ^9^Centre for Addiction and Mental Health, University of Toronto, Toronto, ON, Canada; ^10^Department of Psychiatry, University of Toronto, Toronto, ON, Canada; ^11^Department of Women's and Children's Health, Karolinska Institutet, Stockholm, Sweden

**Keywords:** anorexia nervosa, fMRI, DTI, reward motivation, mesolimbic circuit, ventral tegmental area, nucleus accumbens, bed nucleus of the stria terminalis

## Abstract

Diminished motivation to pursue and obtain primary and secondary rewards has been demonstrated in anorexia nervosa (AN). However, the neurobehavioral mechanisms underlying the behavioral activation component of aberrant reward motivation remains incompletely understood. This work aims to explore this underexplored facet of reward motivation in AN. We recruited female adolescents with AN, restricting type (*n* = 32) and a healthy control group (*n* = 28). All participants underwent functional magnetic resonance imaging (fMRI) while performing a monetary reward task. Diffusion MRI data was also collected to examine the reward motivation circuit's structural connectivity. Behavioral results demonstrated slower speed of reward-seeking behavior in those with AN compared with controls. Accompanying this was lower functional connectivity and reduced white matter structural integrity of the connection between the ventral tegmental area/substantia nigra pars compacta and the nucleus accumbens within the mesolimbic circuit. Further, there was evidence of neurobehavioral decoupling in AN between reward-seeking behavior and mesolimbic regional activation and functional connectivity. Aberrant activity of the bed nucleus of the stria terminalis (BNST) and its connectivity with the mesolimbic system was also evident in AN during the reward motivation period. Our findings suggest functional and structural dysconnectivity within a mesolimbic reward circuit, neurofunctional decoupling from reward-seeking behavior, and abnormal BNST function and circuit interaction with the mesolimbic system. These results show behavioral indicators of aberrant reward motivation in AN, particularly in its activational component. This is mediated neuronally by mesolimbic reward circuit functional and structural dysconnectivity as well as neurobehavioral decoupling. Based on these findings, we suggest a novel circuit-based mechanism of impaired reward processing in AN, with the potential for translation to developing more targeted and effective treatments in this difficult-to-treat psychiatric condition.

## Introduction

Anorexia nervosa (AN) has one of the highest mortality rates among all mental illnesses (1). Classified as an eating disorder, AN is characterized by self-imposed starvation, physical emaciation, an intense fear of weight gain, and a profound disturbance in the way one's shape and weight is experienced (2).

One of the putative disease mechanisms in AN is impairment in reward processing. Disturbances in the experience of reward are evident not only for rewards related to food and eating but extend to other types of reward such as sex and social rewards (3–5). Even premorbidly, diminished novelty seeking has been observed (6), raising the possibility of phenotypic reward-related abnormalities as potential contributors to the development of the disorder. In affected individual with AN, aberrant processing of rewards could manifest as the appearance of an anhedonic state (7–9).

Multiple behavioral and neuroimaging studies in AN using food and non-food reward stimuli have reported impaired reward-related behavior as well as abnormal neural structure and function in reward circuits in the brains of patients with AN (10–18). Based on these findings, a “reward model” of AN (19–22) has been proposed as a compelling framework to study and understand the neurobehavioral mechanisms of symptom genesis in AN.

Reward motivation, or “*wanting*” of a rewarding stimuli/state before obtaining it, is mainly mediated by the mesolimbic system consisting of dopaminergic projections that connect ventral tegmental area\substantia nigra pars compacta (VTA/SNc) regions in the midbrain to the ventral striatum, including nucleus accumbens (NAcc). Reward motivation (or wanting) by itself has been conceptualized to have two components: directional and activational (or “energetic”) (23). The directional aspect deals with valuation and assigning salience to external stimuli for directing behaviors toward, or away from, rewarding or non-rewarding stimuli (24, 25). The activational aspect of motivation is defined by the degree of behavioral activation that an agent exerts to obtain the rewarding stimuli; this can manifest as the speed, vigor, and/or persistence of reward-seeking behaviors' instigation and maintenance (23). Using food- or AN-related stimuli such as those related to weight loss or thinness, several studies examined aberrant salience attribution (related to the directional aspect of reward motivation or wanting) in AN patients (22, 26, 27). However, interrogation of reward motivation in AN from the activational perspective is sparse. Focusing on this unexplored area is particularly important from a clinical perspective. AN patients' capacity to initiate and sustain approach behaviors is critical not only related to food—with relevance for both recovery from the starvation state and maintenance of healthy eating and weight—but also related to pursuing social, monetary, and other rewards that are instrumental to healthy functioning and quality of life.

This work aims to study behavioral and neurobiological mechanisms of reward motivation in AN with a specific focus on the activational elements. To achieve this, we employed a multimodal combination of task-based fMRI and functional and structural connectivity analysis techniques. Our study population consisted of adolescents with AN, restricting type. Adolescents with AN are relatively early in their course of illness and thereby less influenced by chronic ancillary effects of the illness itself, and because of the relevance for future studies that could potentially impact early intervention. The control group consisted of adolescents with mild anxiety symptoms, in order to reduce the confounding effect of this prominent comorbid symptom (in addition to facilitating dimensional explorations of anxiety-related effects across participants, for other planned analyses). Participants engaged in a reward processing fMRI task, from which we measured the speed of reward-seeking behavior as an indicator of the activational facet of reward motivation. We focused on the mesolimbic circuit in our multimodal imaging analyses based on its consistently demonstrated role in reward motivation processes (25, 28, 29).

Our main hypotheses were that adolescents with AN, compared with control adolescents, would exhibit (i) slower speeds of reward-seeking behavior; (ii) lower regional activity of the mesolimbic system (VTA/SNc and NAcc) during the reward motivation period; (iii) lower VTA/SNc-NAcc functional and structural connectivity; and lastly, (iv) neurobehavioral decoupling between the behavioral measure (the speed of reward-seeking behavior) and mesolimbic activity and circuit connectivity during the reward motivation period.

To further understand the neurobehavioral mechanisms of reward motivation in AN, we explored the functional and network dynamics of the bed nucleus of the stria terminalis (BNST) during the reward motivation period. The BNST plays important roles in reward and stress processing and has been implicated in the translational and clinical neuroscience of psychiatric conditions, particularly anxiety disorders and addiction (30, 31). The BNST has functional as well as direct structural connectivity with the NAcc in animals and humans (31–33). Animal studies have demonstrated corticotropin-releasing hormone CRH projections from the BNST to NAcc, which is a putative means by which the BNST may contribute to modulating the NAcc for reward-related motivation and behaviors (34).

## Materials and Methods

### Participants and Clinical Assessments

The UCLA Institutional Review Board approved the study protocol and all methods were performed in accordance with the UCLA Institutional Review Board guidelines and regulations. Informed consents were obtained from parents and/or legal guardians. Recruitment for adolescent participants with AN and control participants was undertaken via local specialized treatment centers, online and community-based advertisements, and campus flyers. AN participants met DSM-5 criteria for AN, restricting type, and had to have recently completed an inpatient, residential or partial hospitalization program (As such, AN participants were partially or fully weight-restored). Controls were included if they scored ≥0.5 STD higher than population norms on the anxiety subscale of the DASS-21, but otherwise not meet criteria for any current DSM-5 disorder. Inclusion criteria for AN were: (1) Ages 10–19; (2) Met DSM-5 criteria for Anorexia Nervosa, Restricting Type, within the previous 6 months; (3) Completed treatment as usual in an inpatient, residential, or partial hospitalization program (2–5 times/week) consisting of psychotherapy and dietary monitoring, within the previous 3 weeks; (4) Participants were either unmedicated or could be taking a serotonin reuptake inhibitor medication at a stable dose for at least 8 weeks at the time of enrollment. They could not be taking any other psychotropic medication aside from a short half-life sedative/hypnotic for insomnia or a short half-life benzodiazepine as needed for anxiety but not exceeding a frequency of 3 doses in 1 week and not taken on the days of the scans. Exclusion criteria for adolescent participants with AN included lifetime Axis I bipolar disorder, lifetime psychotic disorders, lifetime attention-deficit hyperactivity disorder, or current post-traumatic stress disorder. Inclusion criteria for control participants were: (1) females ages 10–19; (2) score at least 0.5 standard deviations higher than population norms on the anxiety portion of the Depression Anxiety Stress Scale (DASS-21) (in order to better dimensionalize relationships between anxiety and brain structure and function across AN and controls). Exclusion criteria for control participants were: (1) any DSM-5 diagnosis [assessed with the MINI KID 7.0.2 (35)]; (2) taking any psychiatric medication. Exclusion criteria for all participants were: (1) current substance abuse or dependence, including nicotine; (2) pathological gambling, as assessed with the South Oaks Gambling Screen; (3) current medical and neurological disorder (i.e., diabetics, hypertension, seizure disorder, migraine headaches, etc.) that requires treatment at the time of the experiment; (4) pregnancy; (5) current major medical disorders that could affect cerebral metabolisms such as diabetes or thyroid disorders; (6) current risk of suicide with a plan and intent; (7) a Children's Depression Rating Scale Revised (CDRS-R) (36) score >75 (extremely ill) or major depressive disorder with psychotic features; (8) ferromagnetic metal implants or devices (electronic implants or devices, infusion pumps, aneurysm clips, metal fragments or foreign bodies, metal prostheses, joints, rods or plates); (9) adjusted BMI ≥ 25 (overweight); (10) visual acuity worse than 20/35 for each eye as determined by Snellen close vision chart (acuity could be met with corrective lenses). Adolescent participants with AN were recruited from the inpatient eating disorder unit at UCLA and from local treatment centers; they were enrolled at the end of their treatment in these settings when they met each treatment center's individual criteria for transitioning to a lower level of care. As such, they were either partially or fully weight-restored.

Clinical evaluations of all participants were performed by licensed psychiatrists or psychologists with clinical experience with this population. Primary or comorbid diagnoses were screened with the Mini-International Neuropsychiatric Interview (MINI KID 7.0.2). Children's Depression Rating Scale™, Revised (CDRS™-R) and depression anxiety stress scales (DASS-21) (37) depression subscale were administrated to measure depression severity in our participants. The Behavioral Inhibition System/Behavioral Activation System (BIS/BAS) (38) was administered, with a focus on the BAS drive scale to assess the strength of motivation each participant had to follow her life's goals. The Hamilton Anxiety Rating Scale (HAMA) (39) was used to measure anxiety that participants had been experiencing in the past week before participating in the study. Furthermore, we asked all participants to rate their level of anxiety right after scanning on a Likert scale (0–10). Eating Disorder Examination (EDE) (40) and Yale-Brown-Cornell Eating Disorder Scale (YBC-EDS) (41) were administered to measure the severity of AN symptoms.

### Task Design

**Figure 1:**
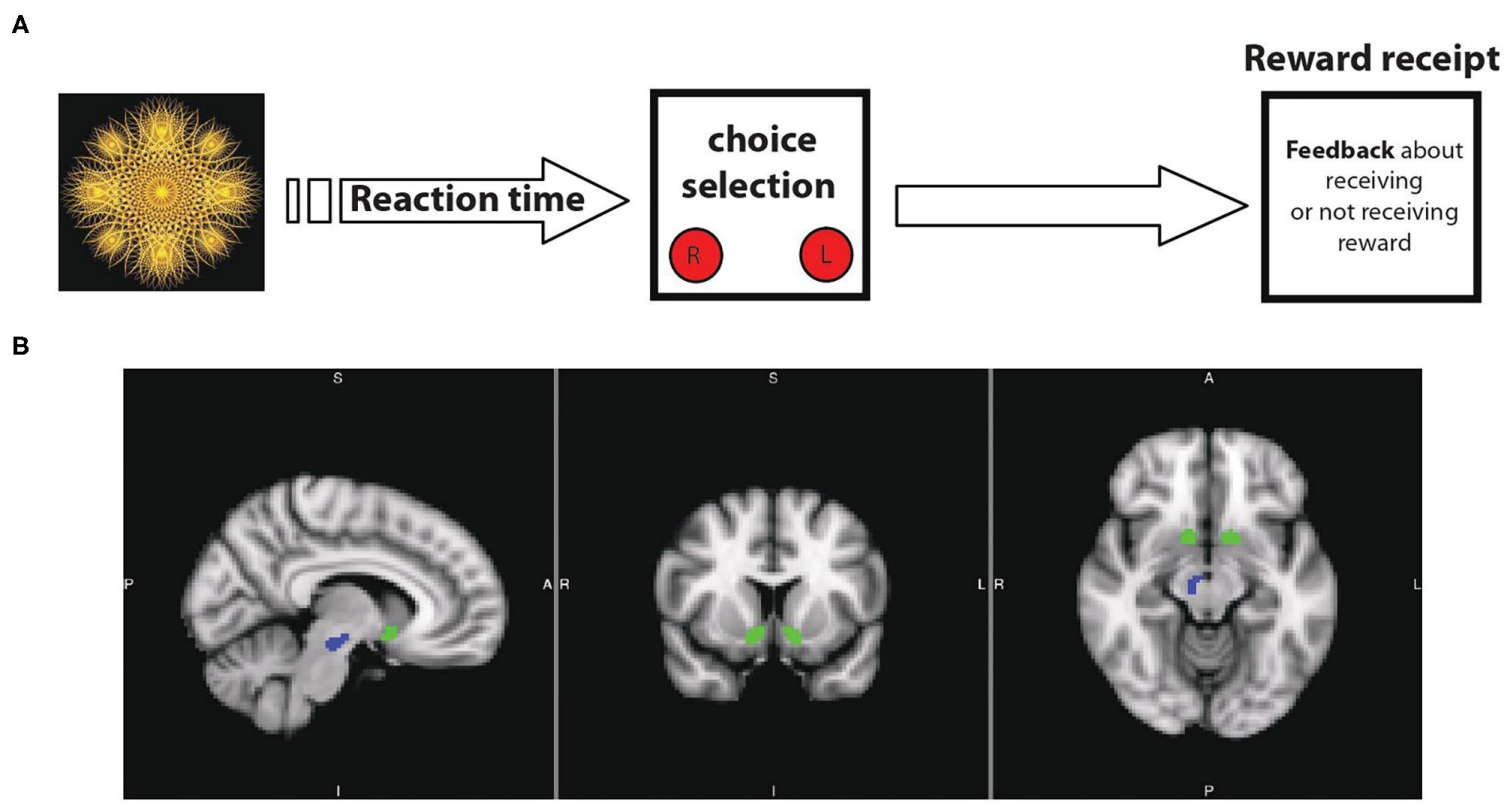
**(A)** Schematic overview of task design: it includes sequential blocks with interval breaks between them. In the beginning of each block, presentation of a fractal image indicates the possibility of obtaining reward after pushing the right or left button. **(B)** Main nodes of mesolimbic circuit mediating motivational behaviors in human: ventral tegmental area\substantia nigra pars compacta (VTA\SNc, in blue) and nucleus accumbens (NAcc, in green).

The design of the task run included sequential sets of stimuli, prompts for responses, and feedback (Figure 1A). Although this task was originally designed to explore reward receipt, we had an idea that some aspect of the task, specifically the reaction time interval (labeled also as reward motivation period), can be utilized to objectively measure the activational aspect of the reward motivation. It should be noted that no information was obtained regarding how much the participants are subjectively motivated to perform the task and obtain the reward. Before the scans, participants were told that they were going to play a game in which they can earn $10 for correct responses. At the beginning of each block, a color fractal image was presented. Participants were instructed to push the right or left button to guess if it belongs in (arbitrarily) “group 1” or “group 2.” After selection, there was a short interval before participants received feedback about guessing correctly or incorrectly and thereby receiving or not receiving the reward (reward expectation period during which participants were presented with neutral words prior to receiving feedback, used for a different part of the experiment.) The chance of receiving a reward in each block was random—50%; therefore, the task did not invoke any implicit or explicit learning.

Each run consisted of 60 interspersed trials optimized for detection of trial-related reward activity (42). Six unique fractal images were used. Each fractal was displayed for 2,000 ms, followed by an inter-stimulus interval randomly jittered between 1,250 and 2,500 ms. Optimized jittering was calculated with optseq2 (43) (https://surfer.nmr.mgh.harvard.edu/optseq). This was followed by the neutral word for 2,000 ms and then a 200 ms fixed inter-stimulus interval before the reward or non-reward feedback, which was displayed for 1,250 ms. Another inter-stimulus interval randomly jittered between 1,250 and 2,500 ms appeared prior to the next fractal. Each trial lasted an average of 6.43 s. Total run length was 498 s.

### Structural, Functional, and Diffusion MRI Data Acquisition

We acquired whole-brain blood oxygenation level dependent (BOLD) fMRI and structural MRI data with a Siemens PRISMA scanner using a 64-channel coil and collected functional echo-planar images (EPI) using: repetition time (RT) 1.0 s; echo time (TE) 33 ms; flip angle, 80°; voxel dimensions, 2 × 2 × 2 mm; multiband acceleration factor, 5; field-of-view 208 mm; 487 measurements; 60 slices. We obtained an MPRAGE T1-weighted image to provide detailed brain anatomy with: TR 2.3 s, TE 2.99 ms, and voxel dimensions 0.08 × 0.08 × 0.08 mm. Diffusion-weighted MRI data were acquired using single-shot spin-echo echo-planar imaging (EPI) (field of view=240 mm; voxel size=1.5 × 1.5 × 1.5, with 0.75 mm gap; TR/TE = 3,222/89 ms and flip angle 89°. We collected 92 slices contiguous axial slices aligned to the AC-PC line along 99 gradient-sensitizing directions with b = 3,000 s/mm^2^, b = 1,500 s/mm^2^, and seven minimally diffusion-weighted scans. We collect this sequence both in the anterior-to-posterior phase encoding direction (AP) and the posterior-to-anterior (PA) direction.

### Region-of-Interest Selection

Our main rationale for focusing on the mesolimbic ROIs is based on extensive and converging animal and human systems neuroscience research showing a fundamental role of the dopaminergic mesolimbic circuit in reward motivation. Mesolimbic ROIs (VTA/SNc and NAcc, Figure 1B) were extracted from Pauli et al., high-resolution probabilistic *in vivo* atlas of human subcortical brain regions (44). Furthermore, we explore the anxiety/stress-related regions (amygdala, anterior cingulate cortex, insula, medial prefrontal cortex, and BNST) for two main reasons: the well-known presence of anxiety/stress dysfunction in AN as well as the direct structural and functional connectivity of those regions with the mesolimbic circuit. We used the Blackford group's BNST segmentation for the BNST ROIs (45) and the Harvard Oxford probabilistic atlas (46) was used for the other ROIs.

### Task-Based FMRI Data Preprocessing and Analysis

Preprocessing of the data was conducted using the FMRIB Software Library (FSL version 6.00). Functional images were realigned to correct for motion-related artifacts using MCFLIRT, filtered with a 90 s high pass filter, spatially smoothed with a 5 mm FWHM Gaussian kernel and resampled to 2 mm cubic isotropic resolution. Both functional and structural images were skull-stripped using BET and co-registered to the MNI space (i.e., MNI152_T1_2mm_brain). Regressors of interest were created by convolving a delta function, representing the motivation period with a canonical (double gamma) hemodynamic response function (HRF). Regressors of no interest included feedback, words, instructions, skipped trials as well as six motion parameters (rotation and translation). Eigenvalues of the BOLD signal were then extracted from the ROIs (above) for further analyses.

### Psychophysiological Interaction (PPI) Connectivity Analysis

We queried whether task-dependent connectivity from the VTA/SNc and from the BNST to the NAcc differed between groups using PPI analyses (47). Mean time courses of the right and left VTA/SNc and the right and left BNST, representing the physiological seeds, were obtained for each participant. These time courses were deconvolved with custom tools [see (48)] regarding the modeling of generalized PPI analysis (48). Four interaction regressions were then produced as the scalar product of these time courses and the time course of the motivation period in the task: the period between the appearance of each fractal image and when the participant made the button-push selection for that image. These were then fit to activation in the bilateral NAcc in a general linear model using the FSL tool FEAT. Age, Pubertal Development Scale (PDS) scores, and subjective anxiety levels after scan were used as covariates for all group comparison analyses. The significance threshold was set at Z > 2.3 and *P* < 0.05, corrected (FLAME 1). We first performed ROI-to-ROI PPI connectivity analyses using voxels averaged in the ROIs. As a *post hoc* analysis, we then tested the averaged-voxel (VTA\SNc or BNST) ROI to the NAcc mask ROI, voxelwise, to explore specific localized connectivity patterns within the NAcc that may not have emerged when all the voxels were averaged across the ROI.

### DTI Data Preprocessing and Tractography Analysis

Diffusion-weighted and T1 images were processed with FMRIB's Software Library (FSL v6.0 www.fmrib.ox.ac.uk/fsl/). For the diffusion data acquisition, reversed phase-encoding directions (AP and PA) resulted in pairs of images with distortions in opposite directions. Prior to tractography, the data were corrected for susceptibility, current-induced distortions, and movement using the FSL topup (49) and eddy (50) tools, respectively. Subsequently, a multi-fiber diffusion model was implemented in FDT, FMRIB's Diffusion Toolbox (51). This model uses Bayesian techniques to estimate a probability distribution function for the principal fiber direction at each voxel, accounting for the possibility of crossing fibers. Two fiber directions were modeled per voxel, using a multiplicative factor of 1 for the prior on the additional modeled fibers and 1,000 iterations before sampling. Diffusion and T1 data were skull stripped using the brain extraction tool (52). Finally, T1 image was segmented (53) to create a cerebrospinal fluid (CSF) mask used later to restrict tractography results to brain voxels only, and linearly registered to diffusion space. Initially the BNST, VTA/SNc and NAcc masks were transformed into each individual's T1 and diffusion imaging space using FLIRT-FNIRT linear and non-linear transformations (54). Then using the FSL probtrackx2 (55) the probabilistic tractography was performed using the BNST and VTA/SNc as seed regions and NAcc area as the target region. Five thousand streamlines were generated from each voxel in the seed area. A curvature threshold of 0.2 was used (~80 degrees) for stopping streamline trajectories. The default 0.5 mm voxel step length, 5,000 samples, and 2,000 steps were used (–steplength 0.5 -P 5000 -S 2000). Fibers with a volume of fraction lower than 0.01 were discarded during tractography using the default value for the subsidiary fiber volume threshold (–fibthresh = 0.01). To avoid artifactual loops, streamlines that loop back on themselves were discarded (–l). Using the —opd and –os2t flags 3D image files were created that contained the number of streamlines that reached each target voxel and seed segmentation maps to each target were derived where the value of each voxel corresponded to the number of streamlines seeded from that voxel reaching the target mask. Each target mask was defined as a waypoint mask in order to discard tracts that do not pass through the target, a termination mask in order to terminate the pathways as soon as they enter the termination mask, and a classification mask in order to quantify connectivity values between the seed and target mask. Finally, for each participant the total number of tracts (streamlines) that were not rejected by the inclusion/exclusion target mask criteria was retrieved. Number of streamlines that reached the target was determined. Then the tensors were determined using DTIFIT producing the FA maps. For each participant, using fslsmaths and fslstats, the average FA values were calculated for each tract.

### Statistical Analyses

The PPI statistical analyses were performed in FSL. Other statistical analyses were performed with SPSS (IBM SPSS Statistics for Windows, Version 24.0. Armonk, NY). ANCOVA was applied for group comparison analyses while controlling for age and PDS score and subjective anxiety levels after the scan. Pearson partial correlations were used to analyze the relationships between imagining variables and reaction time or BAS drive score while controlling for age and PDS scores and anxiety levels after the scan. To assess the interactional role of the BNST in mesolimbic reward motivation neurobehavioral dynamics, we set up an analysis in which the moderation role of the right BNST-to-NAcc connectivity was tested within a simulated (causal) interaction between the right VTA\SNc-NAcc as an independent variable (X) and reaction time as the dependent variable (Y) separately across control and AN group. Moderation analyses were performed by using Hayes's method of ordinary least squares regression-based path analysis, implemented in PROCESS macro (run in SPSS), which also includes bootstrap-based confidence interval calculations (56).

## Results

### Demographics and Clinical Characteristics

Demographic and clinical information for the AN (*n* = 32) and control (*n* = 28) groups are summarized in Table 1. All participants were female. There were statistically significant age and Pubertal Development Score differences between participants with AN and controls. As expected, participants with AN had significantly lower BMI compared to controls (BMI percentile: AN group = 26.0 ± 17.2, control group = 66.4 ± 21.0, t = 7.93 *P* < 0.0001; raw BMI: AN group = 18.2 ± 1.39, control group = 22.3 ± 1.40, t = 8.80, *P* < 0.0001) as well as significantly higher eating disorder symptom severity determined by YBC-EDS and EDE scores. There were no significant differences between AN and control participants on anxiety symptoms as measured by the Hamilton Anxiety Scale (HAMA). Yet, AN scored higher than controls on depression symptom severity as measured by the Child Depression Rating Scale (CDRS™-R) (36) and the depression subscale of the Depression, Anxiety, and Stress Scale (DASS-21) (57). Anxiety ratings after the scan were significantly higher in the AN group compared to controls (HC = 3.1 ± 1.49, AN = 4.4 ± 2.02, *P* = 0.013).

**Table 1:** Demographic and clinical characteristics of patients with AN and control subjects.

	***Adolescent AN***	***Adolescent HC***	***P***
***AGE***	14.8 ± 1.7	16.4 ± 1.9	**0.004**
***Pubertal Development Score***	14.9 ± 3.9	17.7 ± 1.5	**0.001**
***Proportion on medication***	22 out of 32	***–***	–
***BMI percentile***	26.0 ± 17.2	66.4 ± 21.0	**<0.0001**
***Raw BMI***	18.2 ± 1.39	22.3 ± 1.40	**<0.0001**
***Duration of illness (months)***	19.8 ± 19.08	–	–
***YBC-EDS***	24.1 ± 10.9	1.6 ± 3.2	**<0.0001**
***EDE score***	3.1 ± 1.68	0.5 ± 0.66	**<0.0001**
***HAMA***	15.5 ± 10.82	13.8 ± 5.73	0.45
***DASS depression subscale***	18.3 ± 10.6	9.3 ± 6.7	**<0.0001**
***CDRS** **™** **-R***	40.3 ± 16.98	23.3 ± 5.01	**<0.0001**

*AN, Anorexia; HC, healthy control; BMI, Body mass index; YBC-EDS, Yale-Brown-Cornell Eating Disorder Scale; EDE, Eating Disorder Examination; HAMA, Hamilton Anxiety Scale; DASS, depression anxiety stress scales; CDRS™-R, Children's Depression Rating Scale™; Revised. The bold values indicate statistically significant P values (P <0.05)*.

### Mesolimbic Neurobehavioral Mechanisms of Reward Motivation in Control Participants

**Figure 2:**
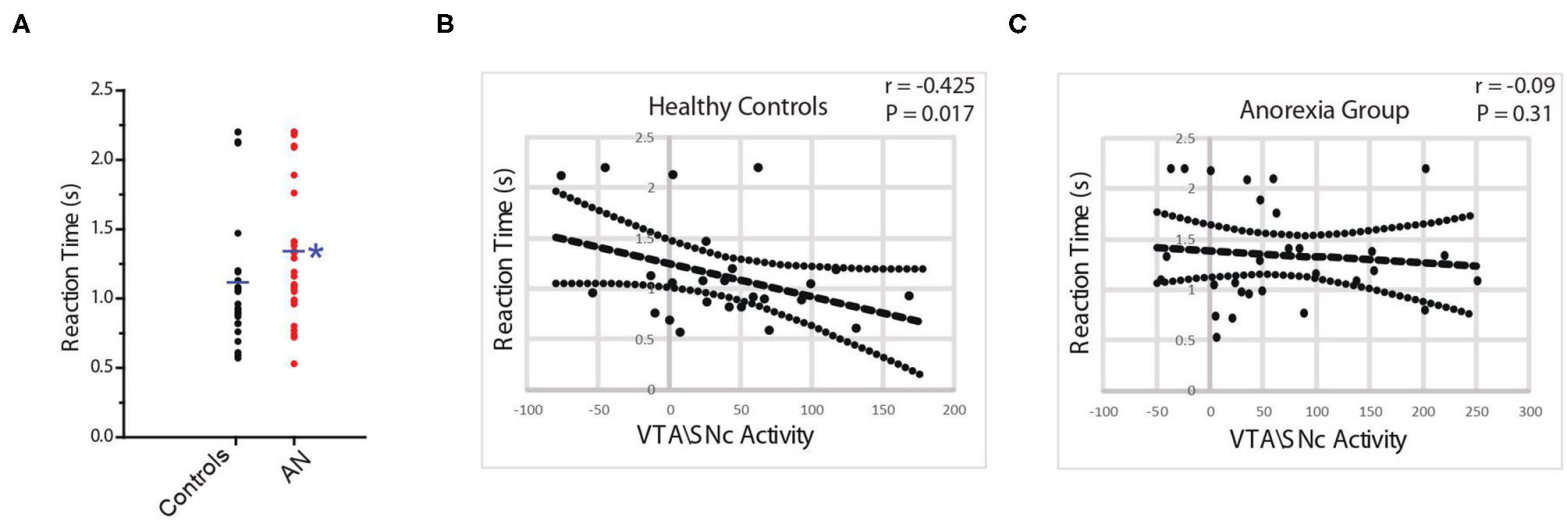
Reaction time (RT) duration is significantly longer in AN participants compared with controls **(A)**. In controls, there is a significant negative correlation between VTA\SNc activity and RT duration **(B)** but not in AN group **(C)**.

**Figure 3:**
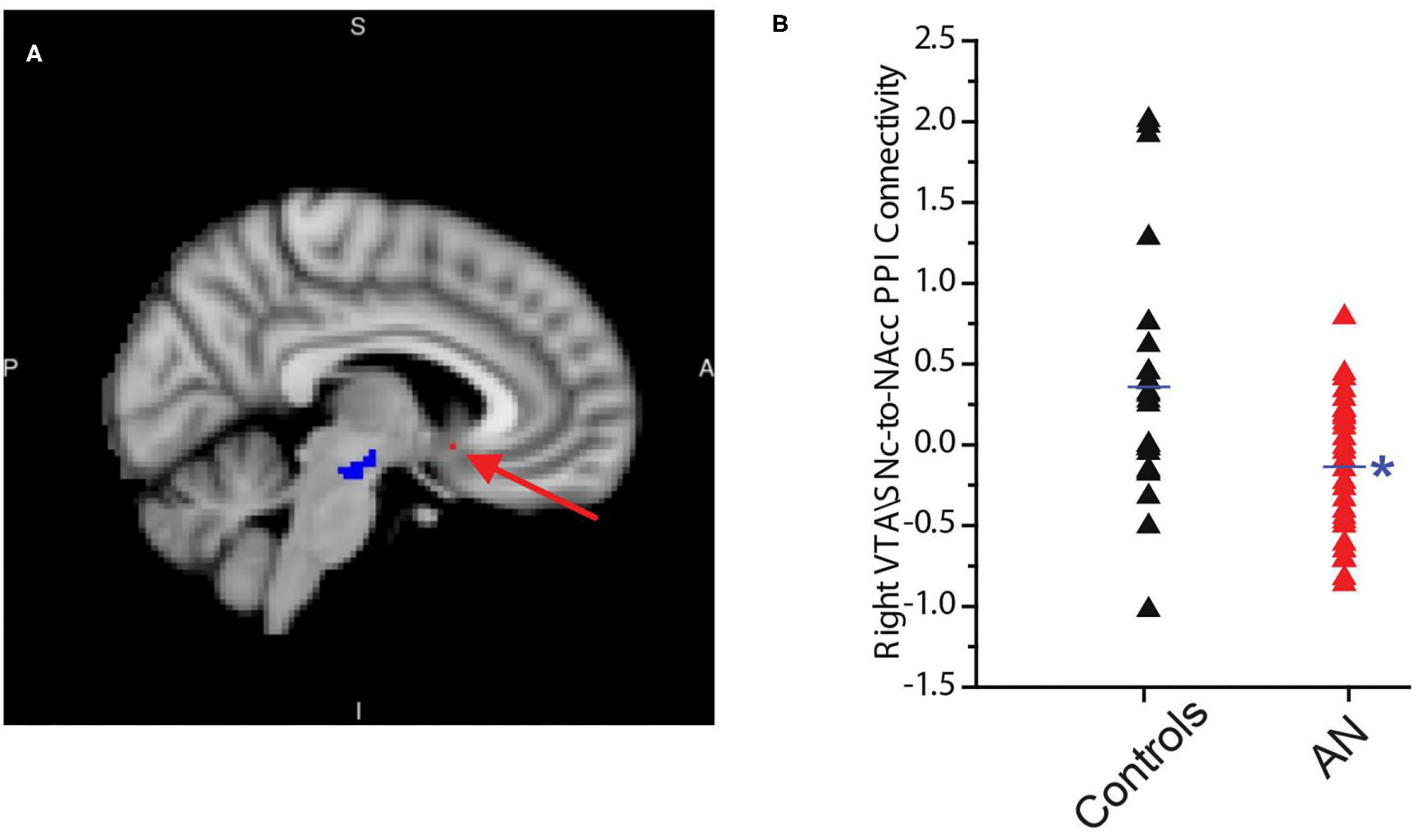
Connectivity of the right ventral tegmental area\substantia nigra pars compacta (VTA\SNc) seed (in blue) with a region in right nucleus accumbens (NAcc) (peak coordinates: 8, 10, −12, in red) is significantly and negatively correlated with duration of reaction time (RT) in controls. No such association exists between the right or left VTA\SNc seeds and any region in the right or left NAcc in the AN group **(A)**. The connectivity of the right VTA\SNc with above-mentioned NAcc area is significantly lower in AN participants compared to controls **(B)**. The * symbol indicates statistically significant.

First, we tested if our task is capable to provide an objective indicator of reward motivation in the participating subjects. In control participants, regional blood oxygen level dependent (BOLD) activity in the bilateral VTA\SNc (but not NAcc) was significantly higher during the reward motivation period compared to the baseline crosshair presentation before starting the task. A significant, negative correlation was found between VTA\SNc level of activity and reaction time (r = −0.54, *p* = 0.005, partial correlation controlling for age, PDS score, and anxiety level after the scan, Figure 2B) during the reward motivation period. Thus, those with greater VTA/SNc activity were faster to push the button. ROI-to-ROI psychophysiological interaction (PPI) functional connectivity analysis showed significantly higher connectivity of the right and left VTA\SNc-to-NAcc during the reward motivation period compared to the baseline (p <0.05, corrected). In the averaged-voxel ROI-to-ROI voxelwise connectivity analysis, connectivity of the right VTA\SNc ROI with the right NAcc (peak coordinates of 8, 10, −12, Figure 3A) was significantly and negatively correlated with reaction time during the reward motivation period. As a *post hoc* analysis, connectivity of the right VTA\SNc to a (3 mm) spherical ROI centered on the NAcc peak coordinate showed a trend level positive correlation with BAS drive scores (r = 0.41, *p* = 0.053, partial correlation controlling for age and PDS score). Based on these neuronal and neurobehavioral findings in control participants, we suggest that the reaction time interval labeled also as “reward motivation period” is associated with the engagement of reward motivation mesolimbic system and therefore give us relevant biomarkers that we can use to objectively explore reward motivation in participants with AN.

### Impaired Mesolimbic Neurobehavioral Coupling in Adolescents With AN

First, we found that the reaction time was significantly longer (slower) in participants with AN compared to controls (HC = 1.1 ± 0.1, AN = 1.3 ± 0.1, F = 4.6, *p* = 0.04, 95% CI: 0.17 to 0.677, observed power = 0.54, ANCOVA controlling for age, PDS score and anxiety level after scan, Figure 2A). No association was found between reaction time and raw BMI value in the AN group (r = 0.21, *p* = 0.32, partial correlation controlling for age, PDS score, and anxiety level after the scan). In the AN group, BOLD activity of the bilateral VTA\SNc and NAcc was higher during the reward motivation period compared to baseline but did not significantly differ from the control group (VTA\SNc: HC = 37.2 ± 58.0, AN = 66.8 ± 89.9, F = 1.90, *p* = 0.17; 95% CI: −14.93 to 80.45, observed power = 0.27, NAcc: HC = −2.8 ± 72.21, AN = 5.4 ± 64.68, F = 0.80, *p* = 0.37; 95% CI: −24.99 to 65.17, observed power = 0.27, ANCOVA controlling for age, PDS score and anxiety level after scan). Unlike controls, no significant correlation was found between the activity level of VTA\SNc and reaction time (r = −0.04, *p* = 0.41, partial correlation controlling for age, PDS score, and anxiety level after the scan, Figure 2C). The ROI-to-ROI connectivity PPI analysis of the right and left VTA\SNc-to-NAcc was significantly higher during the reward motivation period compared to the baseline in the AN group (*p* < 0.05, corrected). Unlike the control group, the averaged-voxel ROI (VTA\SNc)-to-ROI voxelwise (NAcc) PPI analysis did not reveal any clusters in the right or left NAcc showing connectivity with right or left VTA\SNc that significantly correlated with reaction time during the reward motivation period. We found that the connectivity of right VTA\SNc-to-sphere seed (centered around 8, 10, −12 coordinates) during the reward motivation period was significantly lower in the AN group compared to controls (HC = 0.21 ± 0.42, AN = −0.002 ± 0.25, F = 5.021, p = 0.03, 95% CI: −0.477 to −0.026, observed power = 0.59, ANCOVA controlling for age, PDS score and anxiety level after scan, Figure 3B) but showed no significant association with BMI value (r = 0.15, *p* = 0.48, partial correlation controlling for age, PDS score, and anxiety level after the scan. Unlike controls, in the AN group no significant correlation was found between connectivity in the right VTA\SNc-to-sphere and reaction time (r = 0.12, *p* = 0.26, partial correlation controlling for age, PDS score, and anxiety level after scan) or BAS drive score (r = −0.005, *p* = 0.49, partial correlation controlling for age and PDS score).

### Abnormal Structural Integrity of the Mesolimbic Circuit in Adolescents With AN

**Figure 4:**
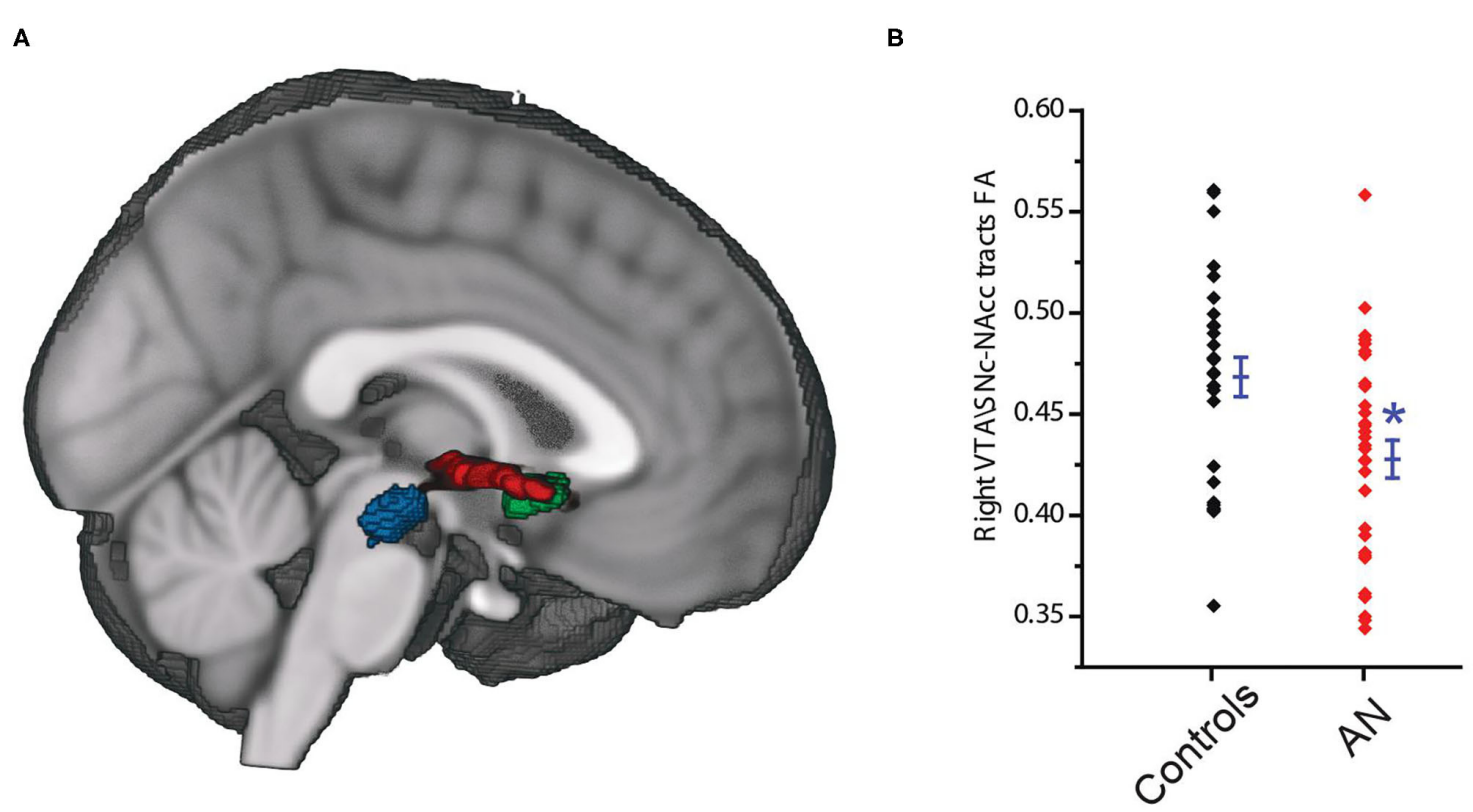
The VTA\SNc (blue) and the NAcc (green) regions of interest, and the connecting white matter tracts (red) **(A)**. Tracts connecting the right VTA\SNc and right NAcc ROIs showed significantly lower lower average fractional anisotropy (FA) in the AN compared to control group **(B)**. The * symbol indicates statistically significant.

The probabilistic tractography analyses (Figure 4A) showed that the tracts connecting the right VTA\SNc and right NAcc ROIs had significantly lower average fractional anisotropy (FA) in AN group compared to controls (AN: 0.42 ± 0.052, HC: 0.46 ± 0.051, *F* = 7.66, *P* = 0.008, 95% CI: −0.072 to −0.011, observed power = 0.77, ANCOVA controlling for age and PDS score, Bonferroni corrected threshold accounting for the right and left hemisphere of *P* < 0.025; Figure 4B). Interestingly, we found a significant and negative association between the right VTA\SNc-NAcc FA and raw BMI value (r = −0.41, *p* = 0.04, partial correlation controlling for age and PDS score). As a follow-up exploratory analysis, we also measured the number of streamlines connecting the right VTA\SNc VTA\SNc and right NAcc ROIs; there were no significant differences between AN and controls (AN = 671 ± 599.9, F = 0.1, p = 0.86, 95% CI: −420.2 to 355.83, observed power = 0.053, ANCOVA controlling for age and PDS score).

### Interaction Between the Bed Nucleus of the Stria Terminalis and Mesolimbic System During the Reward Motivation Period in Adolescents With AN

**Figure 5:**
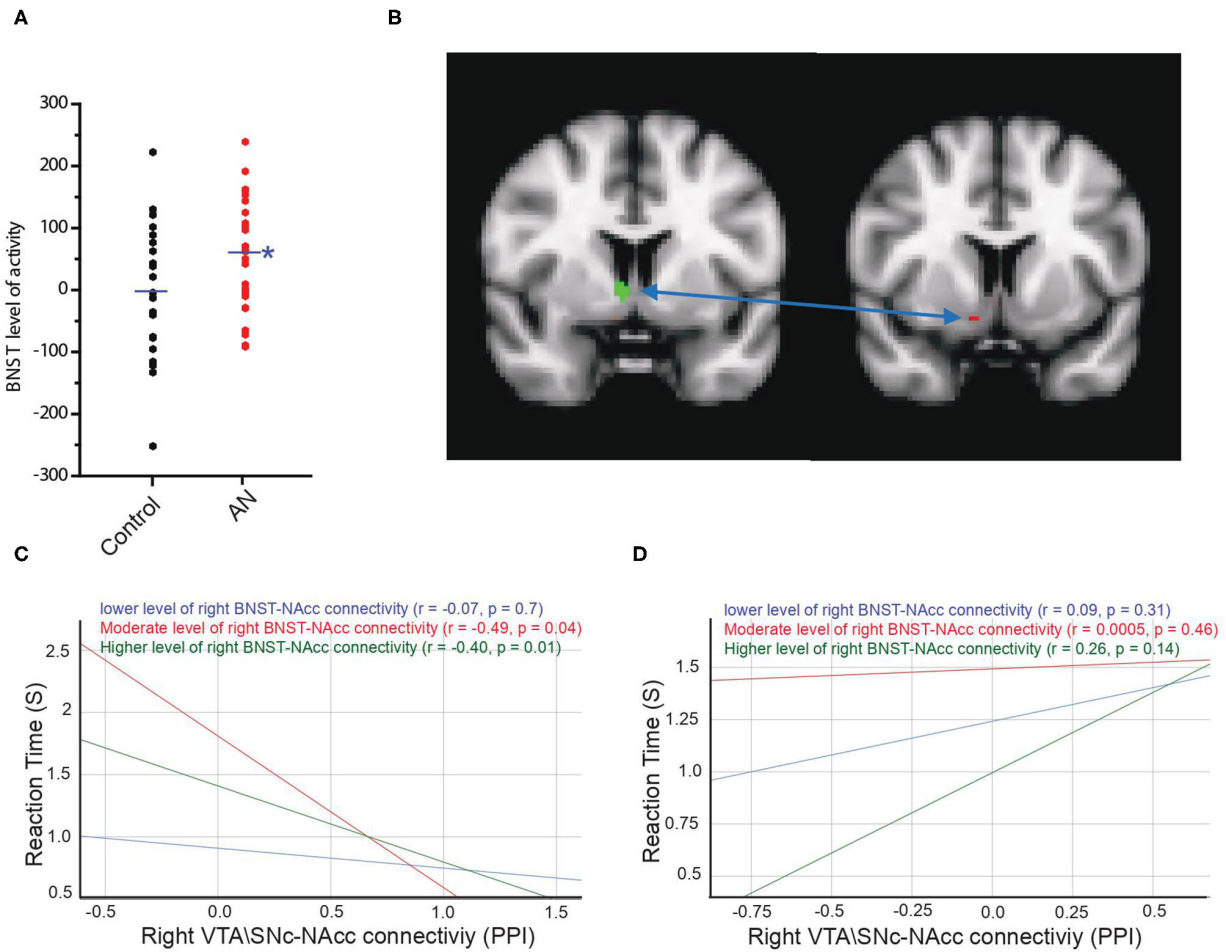
Activity of the bilateral BNST was significantly higher in AN group compared to controls during the reward motivation period **(A)**. Connectivity of the right BNST ROI (in green) with a cluster in right nucleus accumbens (NAcc) (peak coordinates: 10, 8, −12, in red) is significantly lower in AN group compared to controls **(B)**. Moderation results illustrating the association between the right VTA\SNc-NAcc connectivity during reward motivation period as an independent variable (X) and reaction time as the dependent variable (Y) among control **(C)** and AN **(D)** participants with relatively lower (blue), moderate (red) and higher (green) values of the right BNST-NAcc connectivity during reward motivation period. Unlike healthy controls, in AN group there is no significant association between X > Y for any value of the right BNST-NAcc connectivity during reward motivation period. The * symbol indicates statistically significant.

As exploratory analyses, we examined activity in anxiety circuit regions (amygdala, anterior cingulate cortex, insula, medial prefrontal cortex, and BNST) during the reward motivation period. We found that BOLD activity of the bilateral BNST was significantly higher in the AN group compared to controls during the reward motivation period (AN = 60.7 ± 99.15, HC = −2.2 ± 102, F = 5.48, *P* = 0.02, 95% CI: 10.37 to 135.20, observed power = 0.63, ANCOVA controlling for age and PDS score, Figure 5A). The averaged-voxel ROI (BNST)-to-ROI voxelwise (NAcc) PPI connectivity analysis revealed that the connectivity of the right BNST ROI to a cluster in the NAcc (peak coordinates 10, 8, −12) was significantly lower in the AN group compared to controls (Figure 5B). As a follow-up to understand if this BNST-NAcc connectivity affects the neurobehavioral coupling of the VTA\SNc-NAcc connectivity with reaction time, we performed mediation/moderation analyses. We found a significant moderation effect, whereby intermediate and higher values of connectivity between the right BNST and the NAcc ROI (3 mm sphere centered around 10, 8, −12 coordinates) significantly moderated the interaction between the right VTA\SNc-NAcc (the sphere ROI centered around 8, 10, −12 coordinates) connectivity and the reaction time in control participants (intermediate values of VTA\SNc-NAcc connectivity: X effect on Y= −0.51, *p* = 0.04, lower levels for confidence interval (LLCI) = −1.01, upper levels for confidence interval (ULCI) = −0.005; higher values of VTA\SNc-NAcc connectivity: X effect on Y = −0.67, *p* = 0.01, LLCI = −1.18, ULCI = −0.16, Figure 5C). A similar analysis with the AN group, however, did not reveal any significant moderation result for the right BNST-NAcc connectivity (Figure 5D). Further, there was a trend for lower FA in right BNST-NAcc in AN compared to control participants (AN = 0.28 ± 0.040, HC = 0.32 ± 0.037, F = 3.08, *p* = 0.08, 95% CI: −0.46 to 0.003, observed power = 0.40, ANCOVA controlling for age and PDS score).

## Discussion

In this multimodal study, we examined neurobehavioral mechanisms of reward motivation in adolescents with AN. First, the results demonstrated that the task engaged the underlying neurobehavioral dynamics of reward motivation in healthy adolescents; activity of the VTA\SNc and its connectivity with NAcc were both increased compared to baseline and showed significant negative correlations with the behavioral measure of the speed of reward seeking actions. That is, the higher the VTA\SNc activity and the stronger the VTA\SNc-to-NAcc functional connectivity, the faster participants performed the choice selection task that could lead to reward receipt. We also detected a moderation role for BNST-to-NAcc connectivity in the neurobehavioral dynamics of reward motivation. In adolescents with AN, while the regional activity of mesolimbic regions (VTA\SNc and NAcc) did not significantly differ from controls, the functional connectivity between those regions was significantly reduced on the right. Further, in AN, unlike healthy controls, the speed of reward seeking behavior showed no significant correlation with VTA\SNc activity or VTA\SNc-to-NAcc functional connectivity, which suggests a neurobehavioral “decoupling” in patients with AN during reward motivation. In line with these findings, in this same connection the DTI analyses showed evidence of lower white matter integrity in the mesolimbic tract that connect the right VTA\SNc to right Ncc. The exploratory analyses showed higher activity of the BNST but lower connectivity with the NAcc during reward motivation in AN group compared to controls. Further, the BNST did not moderate the underlying neurobehavioral dynamics of reward motivation in AN.

Previous behavioral and neuroimaging studies of reward processing in AN using food- and illness-specific stimuli have shown that AN is associated with an aberrant incentive salience attribution resulting in food (or even sex) rewards being assigned as less implicitly and explicitly wanted (15, 18) or even aversive (58) compared to illness-specific stimuli such as thinness or exercise, which are perceived as more desired (59).

By focusing on the activational aspect of reward motivation, which has been minimally examined in AN, the current study fills an important gap in our understanding of reward processing in AN. Based on our findings, we propose a mesolimbic circuit mechanism that aberrantly impacts the activational aspect of reward motivation in AN. Our results may also help explain the poor general reward, fun, and novelty seeking in individuals with AN. It is possible that the affected individuals might attribute incentive salience normally to monetary reward (a non-food and non-illness-specific stimuli) yet, due to impaired mesolimbic-based neurobehavioral activation, demonstrate abnormalities in the capacity to vigorously approach rewarding stimuli.

Our findings may have relevance in relationship to the proposed “dopamine model” of AN (60). Kaye et al., showed that the concentration of dopamine metabolite homovanillic acid (HVA) in cerebrospinal fluid (CSF) was significantly lower in a group of weight-restored women with restricting-type AN (61). An important question that can be raised is the etiology of low dopamine concentration in the brain regions such as NAcc in the affected patients with AN. Reduction in the general production and metabolism of dopamine due to the malnutrition state from starvation in individuals with AN is one possibility. The findings in the current study of functional and structural dysconnectivity of a mesolimbic circuit involving the VTA and NAcc suggests a complementary (or an alternative) mechanism; specifically, in adolescents with AN, transmission of dopamine from its midbrain VTA\SNc sources to the NAcc may be impaired due to poor within-circuit connectivity. Because the AN participants in this study were recently in a starvation state, this could either represent a lingerign effect of starvation or malnutrition that has not yet resolved since most participants were partially weight-restored, or it could represent an underlying, pre-existing abnormality.

A topic relevant to mesolimbic-based motivational aspects of reward processing in adolescents with AN is the role of mesolimbic dopaminergic function in reward-based learning, particularly considering the developmental dynamics of reward system maturation occurring during the adolescent critical period (62). In an interesting study by DeGuzman et al., reward prediction signaling in the right ventral caudate/nucleus accumbens was found to be significantly higher in underweight adolescents with AN compared to their healthy controls during a monetary reward task, with subsequent normalization after treatment (63). In another study by the same group, female adolescents and young adults with AN performed a sucrose taste classical conditioning task. The reward prediction error signal, described as dopamine-based, was significantly heightened in the caudate head, nucleus accumbens, and insula (64). Furthermore, effective connectivity between the ventral striatum, including the nucleus accumbens, and the hypothalamus was abnormal in AN participants compared to their control group (64). However, the results in these studies are difficult to compare to the current study, as the task in the current study neither includes learning nor reward prediction error computations. Further, mesolimbic circuit connectivity was not specifically performed in those studies. Nevertheless, the current results regarding functional and, especially, structural dysconnectivity of the VTA\SNc-NAcc circuit, may contribute important insights into a potential circuit-based mechanism of aberrant mesolimbic-based reward prediction error computation in AN. One speculation, on the behavioral level, is that slower reaction times in the AN group could be a reflection of delaying or preventing a situation in which there is heighted prediction error, reflecting elevated salience responsiveness (63), which could occur if they do or do not receive the reward. In other words, longer reaction time in AN adolescents may be in attempt to control, and avoid, a possible outcome in which they feel overstimulated.

In a similar and relevant study by Bischoff-Grethe et al., 10 adolescents with restricting-type AN performed a monetary guessing task (65). Interestingly, adolescents with AN showed an exaggerated response to punishment (losses) compared to reward in posterior executive and sensorimotor striatal regions, supporting the notion of AN patients' oversensitivity to criticism and failure. Unlike the findings in our study, the behaviors of participants with AN in terms of reaction time were not significantly different than their control group. However, in that study, the authors focused on brain responses within striatal and cingulate regions after wins and losses, which differs from the approach in the current study in which brain reaction was measured before choice selection.

The findings of this work also need to be discussed in the light of previously reported mesolimbic-based abnormal reward processing in other psychiatric conditions. Very interestingly and nicely Ffitting with our findings, functional alteration abnormalities in the right ventral striatum, including the right nucleus accumbens, was reported in an activation likelihood estimate (ALE) meta-analysis of neuroimaging studies of reward processing in schizophrenia (66) and autism spectrum disorder (67). This suggests that (particularly) right-sided mesolimbic dysfunction might be a possible shared underlying neural mechanism of aberrant reward processing across different psychiatric conditions.

The structural connectivity results in the current study further add to the understanding of reward in AN. VTA\SNc-NAcc tracts belong to the medial forebrain bundle that includes ascending and descending fibers connecting VTA dopaminergic neurons to prefrontal and orbitofrontal (OFC) regions by passing through the NAcc. White matter studies in adolescents with AN are limited and, to the best of our knowledge, no study specifically has tested the VTA\SNc-NAcc connection or the encompassing medial forebrain bundle. Yet, several studies studies reported a reduction in white matter integrity (lower FA) in the fornix, posterior frontal, and parietal areas (68); left superior longitudinal fasciculus (69); corpus callosum, posterior thalamic radiation (70); and the corona radiata (69, 70). In two studies, an increase in FA was reported in the anterior frontal, orbitofrontal, and temporal lobes (68); and bilateral frontal, parietal and temporal areas (71). A possible explanation for the discrepancy between the current results and the studies' findings is that they did not specifically test the VTA\SNc-NAcc connection as a tract of interest. Further, the timing of scanning relative to the starvation state differed; while AN participants in the current study were scanned shortly after acute inpatient or other intensive treatment involving full or partial weight restoration, in the previous studies the scanning was performed at the time of inpatient admission (69, 71), during the course of inpatient (68), or during outpatient (70) treatment. In a longitudinal study, the integrity of white matter tracts, measured by FA, in the reward circuit connecting the NAcc to the lateral OFC was higher in adult patients with AN than healthy controls both while in the starvation state and after weight restoration (16). This was accompanied by higher OFC-to-NAcc inhibitory effective connectivity, suggesting enhanced frontal “top-down” inhibitory control over NAcc. The current study, examining a different part of the reward circuit, showed an opposite direction of abnormalities: decreases in both the white matter integrity and functional connectivity during reward motivation in the VTA-to-NAcc connection. In a separate study, we found evidence in AN of increased structural connectivity (higher volume of white matter fibers compared with controls) in a habitual decision-making circuit consisting of tracts connecting the posterolateral putamen to the premotor/supplementary area (72). Further, there was a significant positive correlation between volume of white matter fibers and the severity of eating disorder-related ritualistic behaviors in the weight-restored AN cohort. A possible mechanism that could explain the findings in the current study as well as the Cha et al. (16) study is that bidirectional dynamics associated with functional utilization of a circuit result in plastic changes in the underlying structural circuit in AN. That is, more (or less) functional utilization of a circuit over time results in higher (or lower) integrity of the involved white matter tracts (73). Based on this notion, we propose a model in which the functional underutilization of the portion of the mesolimbic circuit involved in reward motivation, in regard to consumption of primary food rewards, could result in plastic changes (reduced integrity of fibers) in the underlying white matter tracts. In turn, this could impair the functional capability of the same circuit to be effectively engaged in motivational behaviors toward not only food- and AN-specific stimuli but generalized to other types of reward (e.g., monetary, social, or novel stimuli) in a vicious, circular fashion. Further, the observation that BMI relationships with FA in these tracts are in the oppositive direction provides support for our model that the effect of underutilization of the mesolimbic circuit on white matter tract integrity, rather than a residual metabolic impact of the previous starvation state or the current BMI status, is more likely to account for reduced structural connectivity of the VTA\SNc-NAcc VTA\SNc-NAcc connection in adolescents with AN.

The current study's exploratory analyses uncovered heightened regional activity in the right BNST but decreased right BNST-NAcc connectivity during reward motivation in the AN group. Regarding the abnormally higher regional activation of the bilateral BNST during the reward motivation period, it is possible that this abnormal regional activity could interfere with and adversely impact the BNST network interaction with the NAcc, resulting in a reduction in the right BNST-NAcc PPI connectivity. However, it is challenging to discern the direction of causality—i.e., if an increase in BNST regional activity causes a decrease in BNST-NAcc connectivity or vice versa. The BNST-NAcc functional connectivity in the AN group was not associated with the same modulatory (moderation) function of the BNST in the behavioral reward motivation response as was evident in the control group. Further, the trend for lower FA in this tract in AN suggests a possible reduced structural integrity in this connection. These results suggest that abnormal neuronal activity of the BNST, possibly stress-related, and/or aberrant functional connections of the BNST with the mesolimbic system could be another mechanism that pathologically affects motivation-related behavioral actions in AN, in addition to the primary findings of dysconnectivity within the mesolimbic circuit's functional and structural mechanisms.

Several methodological aspects of this study should be considered. We enrolled adolescents with AN who had recently finished a hospitalization or intensive treatment for the acute phase of their illness, who were thus recently partially or fully weight-restored. While avoiding the confounds of acute starvation, the results cannot necessarily be generalized to acute states of severe malnutrition. In addition, further studies in recovered AN individuals would be useful to help differentiate more fully what is a state-related effect vs. either a trait characteristic or a “scar” of previously being in a severe malnutrition state. The choice of a monetary reward task helped us to determine the activational aspect of reward motivation, which would have been difficult to be accomplish if we had used food or other illness-specific stimuli. Results using food or other illness-specific rewards would be difficult to interpret given the confounding salience attribution for those specific stimuli in AN. Although we used speed (response time) as an indicator of the activational component of motivational behavior, other features such as vigor and persistence, measured as subjects' applied effort to obtain rewards, can be tested using different task such as the Effort-Expenditure for Rewards Task (74). Finally, the cross-sectional nature of the analysis precludes definitive causal inferences from the moderation analyses.

In sum, this study provides empirical evidence of impaired motivational activation in AN, linked to aberrant neurobehavioral decoupling and functional dysconnectivity in the mesolimbic circuit. These neuronal and neurobehavioral abnormalities were accompanied by structural abnormalities in the involved mesolimbic white matter tracts. Furthermore, we uncovered evidence of regional activity and connectivity dysfunction of the BNST in AN, which may represent an additional mechanism that diminishes reward motivation in AN. These findings can be used to refine a neurobiologically and circuit-based model of impaired reward processing in AN, to provide the necessary framework for the development of novel and more effective brain-based treatments in AN.

## Data Availability Statement

The raw data supporting the conclusions of this article will be made available by the authors, without undue reservation.

## Ethics Statement

The studies involving human participants were reviewed and approved by UCLA IRB. The patients/participants provided their written informed consent to participate in this study.

## Author Contributions

RT, JO'D, and JF contributed to the conception and design of the work. RT, D-AM, ET, RR, HW, TM, and WP contributed to the data analysis. RT, NP, AB, SM, JO'D, and JF contributed to the interpretation of the results. RT wrote the main manuscript text. All authors reviewed the manuscript.

## Funding

This work was supported by National Institute of Mental Health (JDF R01MH093535). RT research was supported by National Institute of Mental Health (K23MH116117 and R01MH121089) and Brain & Behavior Research Foundation (NARSAD-27111).

## Conflict of Interest

The authors declare that the research was conducted in the absence of any commercial or financial relationships that could be construed as a potential conflict of interest.

## Publisher's Note

All claims expressed in this article are solely those of the authors and do not necessarily represent those of their affiliated organizations, or those of the publisher, the editors and the reviewers. Any product that may be evaluated in this article, or claim that may be made by its manufacturer, is not guaranteed or endorsed by the publisher.
